# Reliability of Sprint Time and Force—Velocity Profiles During Sprint Acceleration in Elite Rugby Union Players: Inter-Trial Reliability of Linear Encoder, GPS, Timing Gates and Video Analysis

**DOI:** 10.3390/sports14080339

**Published:** 2026-08-06

**Authors:** Samuel Grimbert Le Mer, Adrien Vachon, Iñigo Mujika, Nicolas Berryman, Jean-Benoit Morin, Laurent Bosquet

**Affiliations:** 1 Lab MOVE (UR20296), Faculty of Sport Sciences, University of Poitiers, 86000 Poitiers, France; samuel.grimbert.le.mer@univ-poitiers.fr (S.G.L.M.); avachon@staderochelais.com (A.V.); 2 Stade Rochelais Rugby, Departement Strength and Conditioning, 27 Avenue Maréchal Juin, 17000 La Rochelle, France; 3 Departement of Physiology, Faculty of Medicine and Nursing, University of Basque Country (EHU), 48940 Leioa, Basque Country, Spain; inigo.mujika@inigomujika.com; 4 Département des Sciences de l’Activité Physique, Université du Québec à Montréal (UQAM), Montréal, QC H2X 1Y4, Canada; 5 Inter University Laboratory of Human Movement Biology, EA 7424, University Jean Monnet Saint-Etienne, Lyon 1, University Savoie Mont-Blanc, 42023 Saint-Etienne, France; jeanbenoitmorin@gmail.com

**Keywords:** team sports, measurement, running, force, velocity

## Abstract

This study aimed to test the reliability of measurements obtained using different technologies for sprint time and force–velocity profiles during sprint running. Seventeen elite rugby union players completed three experimental sessions, separated by one week. During each session, players completed two 30 m sprints and measurements were performed simultaneously with a linear encoder, a 10 Hz GPS unit, timing gates and video analysis. Split time (5, 10, 15, 20, 25 and 30 m), maximal velocity (V_MAX_, m·s^−1^) and force–velocity variables (maximal power, P_MAX_, W·kg^−1^; theoretical maximal force, F_0_, N·kg^−1^; theoretical maximal velocity, V_0_, m·s^−1^; maximal ratio of force, RF_MAX_, percentage) from the best sprint of each session were computed. Statistical significance was set at *p* < 0.05 for all analyses. Linear encoder and video analysis showed moderate-to-very-high reliability for sprint time (intraclass correlation coefficient (ICC) = 0.68 to 0.94; standard error of measurement (*SEM*) = 0.95 to 2.59%), while timing gates showed poor-to-high reliability (ICC = 0.23 to 0.85; *SEM* = 1.80 to 7.23%). Linear encoder showed very-high reliability for maximal velocity (ICC = 0.94) and force–velocity variables for P_MAX_ (ICC = 0.90), high reliability for V_0_ (ICC = 0.88) as well as RF_MAX_ (ICC = 0.75), and moderate reliability for F_0_ (ICC = 0.66). Linear encoder (ICC = 0.66 to 0.94) and video analysis (ICC = 0.50 to 0.94) were the most reliable methods to measure sprint times while linear encoder, due to a higher sampling frequency, was the most reliable to establish the force–velocity profile.

## 1. Introduction

Sprint and acceleration are performance-determining qualities in team sports, often contributing to positive game outcomes [1]. In addition to sprint time, performance can be assessed through the mechanical outputs and the force–velocity profile (FVP), which represents a player’s ability to generate propulsive force horizontally over the sprint acceleration spectrum [2]. The FVP can be used to compare athletes, monitor performance over time, interpret the effects of a specific training phase [3] and individualize training content [4].

Measurements of the FVP during sprinting were first performed on motorized sprint treadmills [5]. Then, computations were conducted using force plate systems and 3D motion capture [6]. Even if these methods are considered as “gold standards”, they require expensive equipment and are time-consuming, limiting their regular implementation in the daily training environment. Samozino et al. [2] developed a simple method based on fundamental laws of motion to measure the FVP during a single sprinting acceleration. This method, based on the measurement of velocity–time data, uses a macroscopic inverse dynamics approach applied to the center of mass of the player [2], and allows measurements with affordable devices. Different tools, known as “silver standards”, have also been used to collect spatiotemporal data such as radar and laser devices [6,7,8,9], timing gates [6,10,11,12], satellite-based tracking systems (GPS) [6,8,11,13,14,15,16] and video analysis using the MySprint application [7,10,15].

Recently, technological advances have led to improvements in horizontal linear encoder devices, allowing FVP testing with a high sampling frequency (>200 Hz). Linear encoders have been used by strength and conditioning coaches for both sprint testing [17] and training monitoring [18]. These devices have been validated to measure sprint time on 30 m [19], change of direction [20], and the ground reaction force during resisted sprinting [21]. In addition, linear encoder’s metrological properties for FVP have been studied, by testing the interchangeability with a radar [22], and by testing validity and reliability against GPS, timing gates, radar and laser technologies [6,16,22]. Previous linear encoder studies [6,16,17,18,19,20,21,22] used the first version of the 1080 Sprint device, but it is less portable and less powerful than the most recent one. Moreover, this first device is powered by an electrical outlet, which is not convenient in many field situations. In addition, the only study investigating the reliability of this linear encoder was conducted with 18 participants from different sports and performance levels [6]. Therefore, the reliability of the most recent version of the 1080 Sprint linear encoder including its high sampling frequency (1080 Sprint 2, 1080 Motion, Lidingö, Sweden) needs to be assessed with a homogenous and elite group of athletes in order to subsequently correctly interpret changes in players’ performance.

Overall, the speed–time measurements from which FVP is derived have been shown to be valid and reliable using various tools. However, there is a lack of data concerning the reliability of the newest version of the linear encoder (1080 Sprint 2) with elite rugby union players. The aim of the study was to quantify the test–retest reliability of various methods to measure sprint acceleration metrics: GPS, timing gates, video analysis and linear encoder.

## 2. Materials and Methods

### 2.1. Experimental Approach to the Problem

**Figure 1:**
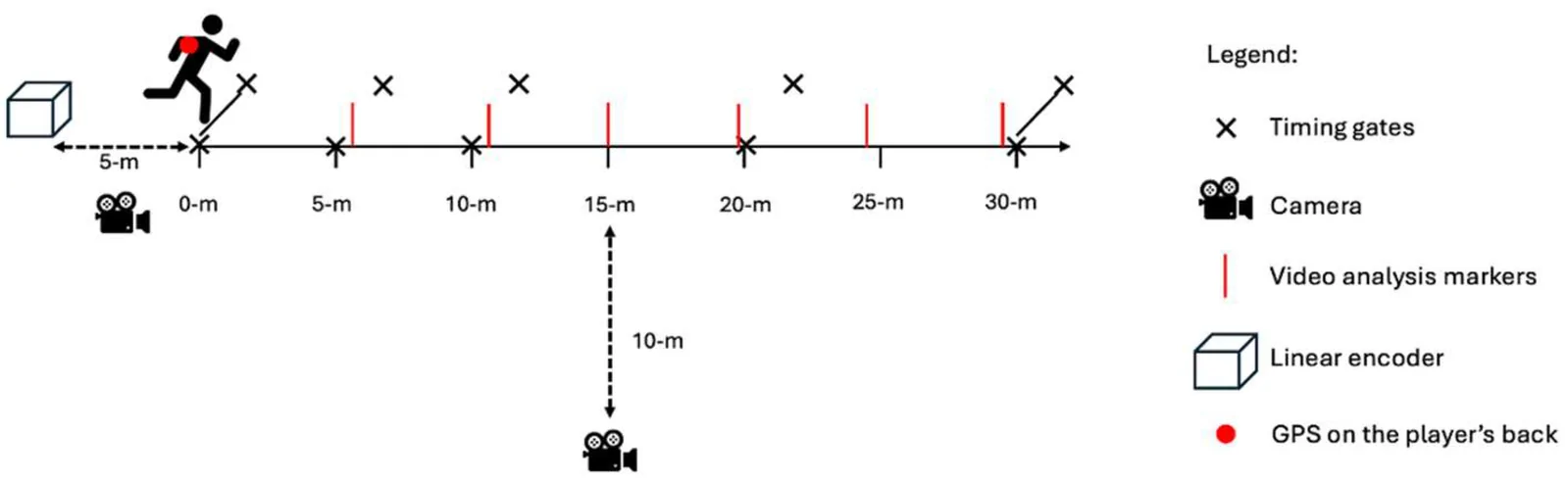
Schematic representation of the testing session experimental setup.

The study was carried out during the 2024/2025 season, during the competitive period. Each player participated in three 30 min sessions separated by 7 days, at the same time of the day. Each session began with a 15 min standardized warm-up including mobility, athletic drills and progressive accelerations. Players were then asked to perform two sprints of 30 m at maximal intensity from a crouching and static starting position, interspersed by 5 min of passive recovery (Figure 1). The start of the sprint was determined as the first movement of the player. From each sprint, an FVP was computed for each device according to Samozino’s method [2], using computations specific to each device’s signal/data. For the linear encoder, the FVP was computed from the software provided by the manufacturer while a custom Excel file was used for the timing gates. A custom-made script on R statistical software (version 2025.09.2+418) was used for the GPS [13] and the video analysis was completed with the MySprint application (version 2.5).

### 2.2. Subject

Twenty-four elite (tier 3 [23]) rugby union players from a Top 14 club (first division of French professional rugby union) participated in this study. Participants were members of the U21 team, playing at the top national level, and training for 12 h per week (including rugby sessions, games, gym sessions). Due to injuries (*n* = 3), selection for the 1st team (*n* = 2), or unavailability (*n* = 2), 7 participants were excluded from the study. The final sample size was 17 players (age = 19.6 ± 0.9 years, height = 1.85 ± 0.1 m, body mass = 98.7 ± 19.6 kg). The experimental session was part of the usual training of the team and subjects were included in the study as they took part in the 3 training sessions. All players were informed of the benefits and risks of the investigation and provided written informed consent to participate in the study which complies with national regulations regarding ethics and data protection (IRB00012476-2024-02-11-350).

### 2.3. Procedures

#### 2.3.1. Linear Encoder

The 1080 Sprint 2 (1080 Motion, Lidingö, Sweden) is a motorized horizontal linear encoder with a sampling frequency of 200 Hz. The device was placed 5 m behind the starting line and set on the No Flying Weight (NFW) mode with a resistance of 1 kg to limit the slack of the cable. The 1 kg resistance did not affect the comparison between the different measurement tools, as all devices measured the same sprint. The cable was connected to a harness attached to the participant’s waist. Power (in W), force (in N) and velocity (in m·s^−1^) were measured continuously during each sprint. FVPs were established for each sprint using the software provided by the manufacturer from the velocity data according to the method described by Samozino et al. [2], with a fitting of the speed–time curves by the exponential model (basis postulate of the method) higher than R^2^ = 0.976 and an average of R^2^ = 0.986.

#### 2.3.2. Global Positioning System

The Catapult Vector S7 (Catapult Innovations, Melbourne, Australia) is a global positioning system that uses GNSS signals (GPS, Glonass and SBAS), with a sampling frequency of 10 Hz. The device was positioned on the participant’s back, along the thoracic spine and between the two scapulas, using a fitted vest provided by the manufacturer. Data were recorded using Openfield (Operator Openfield version 3.13.0, Catapult Innovations, Melbourne, Australia) and exported in CSV format. The GPS positioning quality was 67.92 ± 2.6%, 66.48 ± 3.13% and 63.57 ± 6.89% for the first, second and third sessions, respectively. The average horizontal dilution of precision was 0.85 ± 0.04, 0.86 ± 0.06 and 0.85 ± 0.07 and the number of satellites connected was 14.2 ± 0.5, 14.2 ± 0.7 and 14.6 ± 0.4 for the first, second and third sessions, respectively, which is considered to be within the upper range of good signal quality [24]. FVPs were determined from an Excel file using an application based on the method described by Samozino et al. [2,13].

#### 2.3.3. Timing Gates

The TCi System (Brower Timing Systems, Draper, UT, USA) is a photocell timing system with an error of measurement of 0.01 ms. Photocells were installed on a tripod, placed one meter above the ground on each side of the corridor where the players were sprinting. Timing gates were placed at 0, 5, 10, 20 and 30 m, with 0.5 m offset. The first timing gate was placed 0.5 m after the start line at a lower height to measure the start of the sprint. Overestimation of FVP values is possible due to the delay between the player’s first movement and the triggering of the first timing gate. To avoid this problem, delay between the player’s first movement and the triggering of the first pair of cells was determined by video analysis and totaled with each split time. The camera (iPad 9th generation, Apple Inc., Cupertino, CA, USA) used to determine the time delay was placed 5 m on the side of the starting line on a tripod with a sampling frequency of 60 frames per second. FVP was calculated from split times and anthropometric data using an Excel spreadsheet designed by Jean-Benoît Morin [25] and based on the method described by Samozino et al. [2].

#### 2.3.4. Video Analysis

Sprints were recorded using an iPhone 11 Pro (Apple Inc., Cupertino, CA, USA), placed on a tripod, with a sampling frequency of 60 frames per second. Despite the low sampling frequency compared to the standard of 240 fps, this method is still reliable [26]. The video signal was then analyzed using the MySprint application [7,15]. Each 5 m split time over the 30 m sprint was identified with markers located at 5.57 m (for 5 m), 10.28 m (for 10 m), 15 m (for 15 m), 19.72 m (for 20 m), 24.43 m (for 25 m) and 29.15 m (for 30 m) from the starting line. Athletes ran in the middle of a 1.1 m wide corridor; therefore, the markers were placed at 0.55 m from each athlete, and the camera was set at 10 m from the 15 m marker. FVP was calculated from 5 m split times and anthropometric data according to the method described by Samozino et al. [2].

### 2.4. Statistical Analysis

From each of the three experimental testing sessions, the best sprint based on the fastest sprint time measured by the linear encoder was retained for analysis. The linear encoder and video analysis provided six split times (0–5 m, 5–10 m, 10–15 m, 15–20 m, 20–25 m, 25–30 m), timing gates provided four split times (0–5 m, 0–10 m, 0–20 m and 0–30 m), and GPS provided a continuous measure of time. All devices provided the maximal velocity (V_MAX_, in m·s^−1^). FVPs were established for each device using a simple calculation method based on an inverse dynamics approach applied to the athlete’s center of mass, based on anthropometric and spatiotemporal data [2]. Available parameters are maximal power output (P_MAX_, in W·kg^−1^), theoretical maximal force (F_0_ in N·kg^−1^), theoretical maximal velocity (V_0_, in m·s^−1^), and the maximal ratio of force (RF_MAX_, in%).

Standard statistical methods were used for the calculation of means and standard deviations. Normal Gaussian distribution of the data was verified by the Shapiro–Wilk test and sphericity with the Mauchly test. Independent t-tests were used to observe differences between forwards and backs for the main variables. The magnitude of the difference (effect size, ES) between the groups was assessed using Cohen’s d. The scale proposed by Cohen [27] was used for interpretation, where changes were considered small 0.20 ≤ ES < 0.50; moderate, 0.50 ≤ ES < 0.80; and large, ES ≥ 0.80.

Concerning the reliability, when the assumption of sphericity was not met, the significance of F-ratios was adjusted according to the Greenhouse–Geisser procedure when the epsilon correction factor was <0.75, or according to the Huynh–Feldt procedure when the epsilon correction factor was >0.75. Systematic bias [28] was assessed with an ANOVA for repeated measures. Multiple comparisons were made with the Holm post hoc test. Relative reliability [28] was assessed with the intraclass correlation coefficient (ICC; model 2,1) with confidence intervals at 95%. The absolute reliability was assessed with the standard error of measurement (*SEM*). We considered an ICC over 0.90 very high, between 0.70 and 0.89 high and between 0.50 and 0.69 moderate [29]. The *SEM* was also used to determine the minimum difference to be considered real (*MD*) [28]. The *SEM* was calculated as presented in Equation (1):

(1) $$SEM=\;\sqrt{MS_{E}\;}\;$$

where *MS_E_* is the mean-squared error. The *MD* was calculated based on Equation (2):

(2) $$MD=SEM\times 1.96\times \sqrt{2}$$

where *SEM* is the standard error of measurement computed from Equation (1). According to the results of interventional studies [30], investigating the change in sprint time and FVP variables with sprinters, we considered a percentage of *SEM* and *MD* under 5 as very high absolute reliability, between 5% and 10% as high, between 10% and 15% as moderate and above 15% as poor. Statistical significance was set at *p* < 0.05 for all analyses. All calculations were performed with JASP 0.19.3 (JASP, Amsterdam, The Netherlands).

## 3. Results

Reliability outcomes for sprint time and FVP indices are presented in Table 1 and Table 2, respectively. Systematic biases (*p* < 0.05) were found for the sprint time measured with the linear encoder and video analysis indicating that performance increased from session 1 to session 3 (Table 1). Similar systematic biases were observed for the FVP parameters for all variables measured with the linear encoder and video analysis (Table 2). Effect sizes of the repeated-measure ANOVA are presented in Table 3.

Sprint time showed high-to-very-high relative reliability for the linear encoder (ICC = 0.73 to 0.93) with very high relative and absolute reliability for 25 m and 30 m sprint times (ICC = 0.92; *SEM* = 1.14%; *MD* = 3.17%; ICC = 0.93; *SEM* = 1.22%; *MD* = 3.37%, respectively). Moderate-to-very-high relative reliability was found for the video analysis (ICC = 0.68 to 0.94), with the 5 m sprint time showing the lowest reliability (ICC = 0.68; *SEM* = 2.4%; *MD* = 6.66%). Timing gates showed poor-to-high relative reliability (ICC = 0.23 to 0.85), with poor relative and moderate-to-high absolute reliability for the 5 m and 10 m sprint times (ICC = 0.23; *SEM* = 7.23%; *MD* = 21.14%; ICC = 0.39; *SEM* = 4.26%; *MD* = 11.80%, respectively). V_MAX_ measured with the linear encoder showed very high relative reliability (ICC = 0.94), while other devices displayed high relative reliability (ICC = 0.79 to 0.87).

Concerning FVP variables, the linear encoder showed very high relative and moderate-to-very-high absolute reliability for P_MAX_ (ICC = 0.90; *SEM* = 3.83%; *MD* = 10.61%) and moderate-to-high relative and absolute reliability for F_0_, V_0_ and RF_MAX_ (ICC = 0.66 to 0.88; *SEM* = 4.52 to 2.4%; *MD* = 12.52 to 6.65%). The GPS showed high relative and absolute reliability for P_MAX_ (ICC = 0.85; *SEM* = 3.88%; *MD* = 10.75%), while moderate reliability was found for V_0_. However, poor reliability was found for F_0_ and RF_MAX_ (Table 2). The timing gates showed moderate relative reliability for V_0_ (ICC = 0.63); poor relative reliability for P_MAX_, F_0_ and RF_MAX_ (ICC = 0.11 to 0.48); and poor-to-very-high absolute reliability (*SEM* = 4.71 to 16.53; *MD* = 13.04 to 36.69).

Significant differences were observed for all the variables analyzed with the linear encoder between the forwards and the backs (Table 4). While the video analysis failed to differentiate the backs and forwards for the V_MAX_ and V_0_ (*p* = 0.089; *p* = 0.069, respectively), the GPS and timing gates failed to differentiate the backs and forwards for the F_0_ (*p* = 0.397; *p* = 0.20, respectively).

## 4. Discussion

The aim of this study was to assess the reliability of four devices to measure 30 m sprint time and FVPs during three experimental sessions in elite rugby players. The main results were that (1) linear encoder and video analysis present high and very-high relative and absolute reliability to measure sprint time, and (2) linear encoder was the most reliable device to establish the FVP.

As presented in Table 1 and Table 2, systematic biases were observed on all sprint times and on FVP variables. Participants improved their sprint times across the sessions, as significant differences were observed with the linear encoder and video analysis. Participants also showed increased P_MAX_, F_0_ and RF_MAX_ across the experimental sessions while V_0_ decreased on the third test (Table 3). Even if the participants were trained and used to this type of exercise, these biases show that a learning effect or improvement due to training still occurred. This is in line with studies investigating seasonal variations in the sprint performance, where improvements were observed in sprint performance [31]. Observation of week-to-week variation shows that these qualities evolve over the course of a season, highlighting the importance of rigorous testing methodology and tools and a longer period of familiarization. It is important to mention that systematic biases negatively impact the reliability parameters, but they are inherent to high-level environments.

High-to-very-high relative reliability was observed for sprint time measurements with the linear encoder, whereas a moderate-to-very-high relative reliability was observed with the video analysis. Poor-to-high relative reliability scores were obtained with the timing gates. This is in contrast with studies investigating the inter-trial reliability for sprint times during a session [2,6,9,10,11,13,15,16,22], and studies that tested the between-days reliability [7,12,32]. The inter-trial reliability of timing gates has previously been tested with six 40 m sprints and showed high reliability with a low coefficient of variation (CV) (timing gates: CV = 0.028%) [10]. More recently, the between-days reliability has been tested with two sprints of 20 m sprint separated by 7 days, and the results showed very-high relative reliability for the 10 m and 20 m sprint times (ICC = 0.93; ICC = 0.95, respectively) [12]. These results differ from the current study, as poor relative reliability was observed for the 5 m and 10 m sprint time along with poor absolute reliability, while high relative reliability has been described for the 20 m and 30 m sprint time. The use of a pressure pad [10] or a motion start sensor [12] for the detection of the start of the sprint may explain the differences observed. Meanwhile, the time delay was determined with video analysis in this study and presented poor results. Therefore, we recommend the use of a pressure pad or a motion start sensor to accurately detect the start of the sprint with timing gates, which seems more reliable than the determination of the time delay by video analysis. In this study, the video analysis showed moderate-to-very-high relative reliability, while the sprint time at 5 m showed the lowest reliability. In a previous study, very low CV (CV = 0.027%) [10] and high-to-very-high inter-trial reliability were found for sprint time measurements [7]. Our results show lower reliability than the results presented by Ghigiarelli et al. [7], especially at the beginning of the sprints, due to a lower sampling frequency than other studies (240 fps) [7,10]. The low reliability at the start of the sprint can also be explained by the difference in sprint starting technique between the players. Even with strict instructions, Duthie et al. [33] showed that small adjustments in the starting position and initial motion can influence the 10 m sprint time. The video analysis is reliable for measuring sprint time across the weeks but, to reduce the noise, starting techniques and analyses should be carefully standardized with high sampling frequency. Linear encoder has been validated to measure spatiotemporal data and ground reaction forces during resisted sprints and to assess change of direction performance [20,21]. Rakovic et al. [19] tested the reliability of the linear encoder to measure sprint time across six resisted sprints with elite female handball players, and they showed that sprint time during resisted sprinting is reliable (ICC = 0.81 to 0.95) [19]. Similarly, the present study revealed that sprint time can be measured accurately over a 3-week period. Concerning the maximum velocity measurement, very-high relative reliability was found with the linear encoder. These observations are in line with the study of Fornasier-Santos et al. [6], in which the linear encoder showed the best inter-trial reliability (timing gates: CV = 1.31%; GPS: CV = 1.47%; linear encoder: CV = 1.13%). Together, the results of the current study reveal that the linear encoder is a reliable device to measure sprint time and maximal velocity. The video analysis and the timing gates showed mixed reliability, especially for the first 10 m, most likely because of issues related to the detection of the start of the sprint [10,12] and low sampling frequency.

The relative reliability for the FVP is different between devices. Linear encoder showed moderate-to-very high ICCs while the video analysis revealed moderate-to-high relative reliability. The GPS showed poor-to-high relative reliability while the timing gates revealed poor-to moderate relative reliability. No previous studies have been conducted on the between-days reliability for the FVP, but our results are quite similar with the studies testing the inter-trial reliability. Indeed, in the study by Fornasier-Santos et al. [6], the linear encoder also presented a very-low coefficient of variation. This is an interesting result as we used the most recent version of the linear encoder (1080 Sprint 2). In this study, poor-to-high relative reliability was found for the GPS with a poor relative reliability for F_0_ and RF_MAX_. In addition, moderate and poor absolute reliability were observed for the P_MAX_ and F_0_ estimated with GPS, despite the fact that signal quality data are considered good [24]. Different results were observed in the study by Cormier et al. [11], with very-high relative reliability for the FVP variables, even though the same GPS units were used and present similar number of satellites and lower HDPO. Differences can be observed in the computation of the FVP between the two studies. First, a velocity threshold (>0.2 m·s^−1^) was used to detect the start of the sprint in the current study, while no such threshold was implemented by Cormier et al. [11]. However, the participants were instructed to stand still 5 s before starting their sprint [11]. Moreover, barometric pressure, wind velocity and temperature were collected for the computation of aerodynamic forces [11]. In contrast, the current study only used the participants’ body mass for the computation. Stockdale et al. [15] also investigated the inter-trial reliability and reported poor reliability with the GPS for F_0_ (ICC = 0.064) and V_0_ (ICC = 0.437) when using the same threshold (>0.2 m·s^−1^) but with different GPS units. Therefore, in addition to GPS units with a high sampling frequency, a lower velocity threshold to detect the beginning of the sprint and the computation of aerodynamic forces seem important to optimize reliability [2].

### 4.1. Limitations

This study has limitations. The high-performance environment in which the study was conducted meant that the sample size was limited and that only two sprints could be carried out per experimental session. Furthermore, biases were observed between the tests due to the 7-day delay between sessions. Sprint performance can fluctuate across the week and during a season [31], which may have affected the reliability. Also, a higher sampling frequency of the camera during the video analysis would have been necessary to ensure better reliability. These methodological discrepancies might represent sources of variability but are inherent to these technologies and their ecological implementation in the daily training environment of elite athletes.

### 4.2. Practical Applications

- The linear encoder used in this study presents the best choice, as high test–retest relative and absolute reliability was found for measuring sprint time and high-to-very high relative reliability for the FVP.
- Video analysis is a more affordable option, but it is crucial to use the highest possible sampling frequency.
- GPS and timing gates are less reliable especially for the start of the sprint. We recommend using a velocity threshold lower than 0.2 m·s^−1^ and including air resistance for the GPS and pressure pads or motion start sensors for the timing gates.
- Linear encoder showed differences between forwards and backs for all variables, and while differences were not observed on all variables for the other devices.

## 5. Conclusions

This study demonstrated that the most recent version of the 1080 Motion linear encoder presents high reliability for measuring sprint time and force–velocity variables, most likely due to a high sampling frequency. Other devices tested (GPS, timing gates, video analysis) present lower reliability, showing the influence of the human intervention in setting up the equipment and processing the data. The results presented in this study are specific to the context of a high-level training environment and to this sample of rugby athletes.

## Figures and Tables

**Table 1:** Sprint time reliability.

Parameters	Devices	Test 1 (Mean ± SD)	Test 2 (Mean ± SD)	Test 3 (Mean ± SD)	ICC (95% CI)	*SEM* (%)	*MD* (%)
V_MAX_ (m·s^−1^)	Linear encoder	8.25 ± 0.49	8.29 ± 0.48	8.29 ± 0.46	0.94 (0.87; 0.98)	1.43	3.96
	GPS	8.15 ± 0.46 ^c^	8.16 ± 0.48 ^c^	8.09 ± 0.43 ^c^	0.87 (0.78; 0.96)	2.02	5.60
	Timing gates	8.02 ± 0.50 ^c^	8.14 ± 0.44 ^c^	8.05 ± 0.41 ^c,e^	0.79 (0.60; 0.91)	2.57	7.12
	Video analysis	7.95 ± 0.51 ^c,d^	8.03 ± 0.44 ^a,c^	7.86 ± 0.45 ^c,d^	0.86 (0.73; 0.94)	1.99	5.52
0–5 m (s)	Linear encoder	1.23 ± 0.07	1.24 ± 0.08	1.20 ± 0.06 ^b^	0.73 (0.47; 0.87)	2.59	7.18
	Timing gates	1.28 ± 0.12	1.35 ± 0.11 ^c^	1.30 ± 0.12 ^c^	0.23 (−0.07; 0.54)	7.63	21.14
	Video analysis	1.33 ± 0.06 ^c^	1.33 ± 0.06 ^c^	1.29 ± 0.07 ^b,c^	0.68 (0.37; 0.83)	2.4	6.66
0–10 m (s)	Linear encoder	1.99 ± 0.10	1.99 ± 0.10	1.96 ± 0.09	0.76 (0.58; 0.90)	2.26	6.26
	Timing gates	2.09 ± 0.08 ^c^	2.14 ± 0.13 ^c^	2.08 ± 0.14 ^c^	0.39 (0.08; 0.67)	4.26	11.80
	Video analysis	2.11 ± 0.09 ^a,c^	2.09 ± 0.09 ^c^	2.06 ± 0.09 ^c^	0.82 (0.60; 0.91)	1.52	4.21
0–15 m (s)	Linear encoder	2.67 ± 0.12	2.67 ± 0.13	2.63 ± 0.12 ^b^	0.84 (0.69; 0.94)	1.68	4.67
	Video analysis	2.80 ± 0.11 ^a,c^	2.77 ± 0.11 ^c^	2.75 ± 0.13 ^c^	0.88 (0.71; 0.94)	1.14	3.16
0–20 m (s)	Linear encoder	3.31 ± 0.15 ^a^	3.30 ± 0.15	3.26 ± 0.15	0.89 (0.76; 0.95)	1.36	3.77
	Timing gates	3.40 ± 0.12 ^c,e^	3.44 ± 0.17 ^c^	3.39 ± 0.19 ^c^	0.74 (0.51; 0.88)	2.45	6.80
	Video analysis	3.46 ± 0.14 ^b,c^	3.43 ± 0.14 ^c^	3.41 ± 0.15 ^c^	0.88 (0.80; 0.96)	1.30	3.61
0–25 m (s)	Linear encoder	3.93 ± 0.18 ^a^	3.92 ± 0.19	3.88 ± 0.18	0.92 (0.83; 0.97)	1.14	3.17
	Video analysis	4.09 ± 0.18 ^b,c^	4.06 ± 0.17 ^c^	4.05 ± 0.18 ^c^	0.92 (0.85; 0.97)	1.10	3.05
0–30 m (s)	Linear encoder	4.53 ± 0.22 ^a^	4.52 ± 0.22	4.49 ± 0.21	0.93 (0.86; 0.97)	1.21	3.37
	Timing gates	4.65 ± 0.19 ^c,e^	4.68 ± 0.23 ^c^	4.63 ± 0.24 ^c,e^	0.85 (0.72; 0.94)	1.80	4.98
	Video analysis	4.72 ± 0.22 ^c^	4.68 ± 0.20 ^c^	4.69 ± 0.21 ^c^	0.94 (0.87; 0.98)	0.95	2.64

V_MAX_ (m·s^−1^) = maximal velocity; ICC = intraclass correlation coefficient; CI = confidence interval; *SEM* = standard error of measurement; *MD* = minimum difference to be considered real; ^a^ different from test 3; ^b^ different from the two other tests; ^c^ different from the linear encoder; ^d^ different from the GPS; ^e^ different from the video analysis.

**Table 2:** Force–velocity variable reliability.

Parameters	Devices	Test 1 (Mean ± SD)	Test 2 (Mean ± SD)	Test 3 (Mean ± SD)	ICC (95% CI)	*SEM* (%)	*MD* (%)
P_MAX_ (W·kg^−1^)	Linear encoder	16.48 ± 2.31 ^a^	16.49 ± 1.97	16.98 ± 2.17	0.90 (0.80–0.96)	3.83	10.61
	GPS	14.56 ± 1.48 ^c^	14.70 ± 1.39 ^c^	14.97 ± 1.60 ^c^	0.85 (0.71; 0.94)	3.88	10.75
	Timing gates	18.01 ± 2.02 ^c,d,e^	17.09 ± 3.57 ^d^	18.53 ± 4.03 ^d^	0.49 (0.19; 0.74)	13.19	36.56
	Video analysis	16.73 ± 2.13 ^a,d^	17.19 ± 2.13 ^d^	17.89 ± 2.45 ^c,d^	0.80 (0.61; 0.91)	5.05	13.99
F_0_ (N·kg^−1^)	Linear encoder	7.41 ± 0.61	7.42 ± 0.56	7.70 ± 0.67	0.66 (0.40; 0.84)	4.52	12.52
	GPS	6.70 ± 0.33 ^c^	6.68 ± 0.47 ^c^	6.94 ± 0.46 ^c^	0.07 (−0.18; 0.40)	6.04	16.73
	Timing gates	8.66 ± 0.88 ^c,d^	8.02 ± 1.61 ^d^	8.85 ± 1.82 ^c,d^	0.11 (−0.15; 0.44)	16.54	45.86
	Video analysis	8.18 ± 0.73 ^c,d^	8.33 ± 0.76 ^c,d^	8.87 ± 0.91 ^b,c,d^	0.50 (0.17; 0.73)	6.13	17.0
V_0_ (m·s^−1^)	Linear encoder	8.87 ± 0.76	8.89 ± 0.57	8.81 ± 0.52	0.88 (0.76; 0.95)	2.47	6.86
	GPS	8.68 ± 0.71 ^c^	8.84 ± 0.93	8.55 ± 0.40 ^c^	0.62 (0.40; 0.84)	4.99	13.83
	Timing gates	8.33 ± 0.63 ^c,d,e^	8.53 ± 0.55 ^c,e^	8.38 ± 0.51 ^c,e^	0.63 (0.36; 0.83)	4.06	11.27
	Video analysis	8.16 ± 0.55 ^c,d^	8.24 ± 0.48 ^a,c,d^	8.06 ± 0.48 ^c,d^	0.86 (0.72; 0.94)	2.12	5.89
RF_MAX_ (%)	Linear encoder	49.50 ± 2.79	49.43 ± 2.33	50.48 ± 2.54	0.75 (0.54; 0.89)	2.40	6.65
	GPS	41.16 ± 2.45 ^c^	41.63 ± 2.51 ^c^	42.64 ± 2.20 ^c^	0.34 (0.10; 0.68)	4.60	12.74
	Timing gates	45.82 ± 1.98 ^c,d,e^	44.41 ± 3.16 ^c,d,e^	45.59 ± 3.59 ^c,d,e^	0.48 (0.18; 0.73)	4.71	13.04
	Video analysis	52.47 ± 2.76 ^a,c,d^	52.41 ± 3.68 ^c,d^	54.24 ± 2.99 ^c,d^	0.55 (0.25; 0.78)	3.80	10.54

P_MAX_ (W·kg^−1^) = maximal power output; F_0_ (N·kg^−1^) = theoretical maximal force; V_0_ (m·s^−1^) = theoretical maximal velocity; RF_MAX_ (%) = maximal ratio of force; ICC = intraclass correlation coefficient; CI = confidence interval; *SEM* = standard error of measurement; *MD* = minimum difference to be considered real; ^a^ different from test 3; ^b^ different from the two other tests; ^c^ different from the linear encoder; ^d^ different from the GPS; ^e^ different from the video analysis.

**Table 3:** Effect sizes for the repeated-measure ANOVA.

Parameters	Devices	Test 1 (Mean ± SD)	Test 2 (Mean ± SD)	Test 3 (Mean ± SD)	Effect Sizes (1 vs. 2; 1 vs. 3; 2 vs. 3)		
V_MAX_ (m·s^−1^)	Linear encoder	8.25 ± 0.49	8.29 ± 0.48	8.29 ± 0.46	-	-	-
	GPS	8.15 ± 0.46 ^c^	8.16 ± 0.48 ^c^	8.09 ± 0.43 ^c^	-	-	-
	Timing gates	8.02 ± 0.50 ^c^	8.14 ± 0.44 ^c^	8.05 ± 0.41 ^c,e^	-	-	-
	Video analysis	7.95 ± 0.51 ^c,d^	8.03 ± 0.44 ^a,c^	7.86 ± 0.45 ^c,d^	-	-	**0.36**
0–5 m (s)	Linear encoder	1.23 ± 0.07	1.24 ± 0.08	1.20 ± 0.06 ^b^	-	**0.45**	**0.55**
	Timing gates	1.28 ± 0.12	1.35 ± 0.11 ^c^	1.30 ± 0.12 ^c^	-	-	-
	Video analysis	1.33 ± 0.06 ^c^	1.33 ± 0.06 ^c^	1.29 ± 0.07 ^b,c^	-	**0.63**	**0.52**
0–10 m (s)	Linear encoder	1.99 ± 0.10	1.99 ± 0.10	1.96 ± 0.09	-	-	-
	Timing gates	2.09 ± 0.08 ^c^	2.14 ± 0.13 ^c^	2.08 ± 0.14 ^c^	-	-	-
	Video analysis	2.11 ± 0.09 ^a,c^	2.09 ± 0.09 ^c^	2.06 ± 0.09 ^c^	-	**0.52**	-
0–15 m (s)	Linear encoder	2.67 ± 0.12	2.67 ± 0.13	2.63 ± 0.12 ^b^	-	**0.35**	**0.32**
	Video analysis	2.80 ± 0.11 ^a,c^	2.77 ± 0.11 ^c^	2.75 ± 0.13 ^c^	-	**0.42**	-
0–20 m (s)	Linear encoder	3.31 ± 0.15 ^a^	3.30 ± 0.15	3.26 ± 0.15	-	**0.28**	-
	Timing gates	3.40 ± 0.12 ^c,e^	3.44 ± 0.17 ^c^	3.39 ± 0.19 ^c^	-	-	-
	Video analysis	3.46 ± 0.14 ^b,c^	3.43 ± 0.14 ^c^	3.41 ± 0.15 ^c^	**0.25**	**0.33**	-
0–25 m (s)	Linear encoder	3.93 ± 0.18 ^a^	3.92 ± 0.19	3.88 ± 0.18	-	**0.26**	-
	Video analysis	4.09 ± 0.18 ^b,c^	4.06 ± 0.17 ^c^	4.05 ± 0.18 ^c^	**0.21**	**0.25**	-
0–30 m (s)	Linear encoder	4.53 ± 0.22 ^a^	4.52 ± 0.22	4.49 ± 0.21	-	**0.21**	-
	Timing gates	4.65 ± 0.19 ^c,e^	4.68 ± 0.23 ^c^	4.63 ± 0.24 ^c,e^	-	-	-
	Video analysis	4.72 ± 0.22 ^c^	4.68 ± 0.20 ^c^	4.69 ± 0.21 ^c^	-	-	-
P_MAX_ (W·kg^−1^)	Linear encoder	16.48 ± 2.31 ^a^	16.49 ± 1.97	16.98 ± 2.17	-	**−2.68**	-
	GPS	14.56 ± 1.48 ^c^	14.70 ± 1.39 ^c^	14.97 ± 1.60 ^c^	-	-	-
	Timing gates	18.01 ± 2.02 ^c,d,e^	17.09 ± 3.57 ^d^	18.53 ± 4.03 ^d^	-	-	-
	Video analysis	16.73 ± 2.13 ^a,d^	17.19 ± 2.13 ^d^	17.89 ± 2.45 ^c,d^	-	**−0.52**	-
F_0_ (N·kg^−1^)	Linear encoder	7.41 ± 0.61	7.42 ± 0.56	7.70 ± 0.67	-	-	-
	GPS	6.70 ± 0.33 ^c^	6.68 ± 0.47 ^c^	6.94 ± 0.46 ^c^	-	-	-
	Timing gates	8.66 ± 0.88 ^c,d^	8.02 ± 1.61 ^d^	8.85 ± 1.82 ^c,d^	-	-	-
	Video analysis	8.18 ± 0.73 ^c,d^	8.33 ± 0.76 ^c,d^	8.87 ± 0.91 ^b,c,d^	-	**−0.85**	**−0.67**
V_0_ (m·s^−1^)	Linear encoder	8.87 ± 0.76	8.89 ± 0.57	8.81 ± 0.52	-	-	-
	GPS	8.68 ± 0.71 ^c^	8.84 ± 0.93	8.55 ± 0.40 ^c^	-	-	-
	Timing gates	8.33 ± 0.63 ^c,d,e^	8.53 ± 0.55 ^c,e^	8.38 ± 0.51 ^c,e^	-	-	-
	Video analysis	8.16 ± 0.55 ^c,d^	8.24 ± 0.48 ^a,c,d^	8.06 ± 0.48 ^c,d^	-	-	**0.36**
RF_MAX_ (%)	Linear encoder	49.50 ± 2.79	49.43 ± 2.33	50.48 ± 2.54	-	-	-
	GPS	41.16 ± 2.45 ^c^	41.63 ± 2.51 ^c^	42.64 ± 2.20 ^c^	-	-	-
	Timing gates	45.82 ± 1.98 ^c,d,e^	44.41 ± 3.16 ^c,d,e^	45.59 ± 3.59 ^c,d,e^	-	-	-
	Video analysis	52.47 ± 2.76 ^a,c,d^	52.41 ± 3.68 ^c,d^	54.24 ± 2.99 ^c,d^	-	**−0.56**	-

V_MAX_ (m·s^−1^) = maximal velocity; P_MAX_ (W·kg^−1^) = maximal power output; F_0_ (N·kg^−1^) = theoretical maximal force; V_0_ (m·s^−1^) = theoretical maximal velocity; RF_MAX_ (%) = maximal ratio of force; ^a^ different from test 3; ^b^ different from the two other tests; ^c^ different from the linear encoder; ^d^ different from the GPS; ^e^ different from the video analysis; bold = effect sizes of significant results from post hoc comparison; - = no significant results.

**Table 4:** Between-group differences for sprint time and force–velocity variables.

Variables	All Players (*n* = 17)	Forwards (*n* = 11)	Backs (*n* = 6)	Between-Group Differences (*p*; ES)
**V_MAX_ (m·s^−1^)**				
Linear encoder	8.27 ± 0.47	8.13 ± 0.39	8.55 ± 0.48	***p* = 0.001**; ES = 0.99
GPS	8.13 ± 0.46	7.92 ± 0.35	8.38 ± 0.50	***p* < 0.001**; ES = 1.13
Timing gates	8.07 ± 0.45	7.95 ± 0.37	8.28 ± 0.51	***p* = 0.011**; ES = 0.77
Video analysis	7.94 ± 0.46	7.86 ± 0.40	8.09 ± 0.40	*p* = 0.089; ES = 0.51
**0–10 m (s)**				
Linear encoder	1.98 ± 0.10	2.01 ± 0.08	1.93 ± 0.09	***p* = 0.001**; ES = 0.99
Timing gates	2.10 ± 0.12	2.13 ± 0.09	2.05 ± 0.18	***p* = 0.010**; ES = 0.78
Video analysis	2.08 ± 0.09	2.13 ± 0.07	2.01 ± 0.07	***p* < 0.001**; ES = 1.72
**0–20 m (s)**				
Linear encoder	3.29 ± 0.15	3.34 ± 0.13	3.20 ± 0.14	***p* < 0.001**; ES = 1.04
Timing gates	3.41 ± 0.16	3.46 ± 0.12	3.32 ± 0.19	***p* = 0.001**; ES = 1.01
Video analysis	3.43 ± 0.14	3.49 ± 0.11	3.34 ± 0.14	***p* < 0.001**; ES = 1.23
**0–30 m (s)**				
Linear encoder	4.51 ± 0.21	4.58 ± 0.19	4.39 ± 0.20	***p* < 0.001**; ES = 1.03
Timing gates	4.65 ± 0.22	4.73 ± 0.17	4.52 ± 0.24	***p* < 0.001**; ES = 1.03
Video analysis	4.70 ± 0.21	4.77 ± 0.17	4.56 ± 0.21	***p* < 0.001**; ES = 1.16
**P_MAX_ (W·kg^−1)^**				
Linear encoder	16.65 ± 2.13	15.86 ± 1.42	18.11 ± 2.46	***p* < 0.001**; ES = −1.22
GPS	14.57 ± 1.48	14.05 ± 1.07	15.43 ± 1.70	***p* = 0.001**; ES = −1.03
Timing gates	17.87 ± 3.31	16.83 ± 2.22	19.78 ± 4.12	***p* = 0.002**; ES = −0.98
Video analysis	17.27 ± 2.25	16.23 ± 1.48	19.16 ± 2.21	***p* < 0.001**; ES = −1.66
**F_0_ (N·kg^−1^)**				
Linear encoder	7.51 ± 0.62	7.31 ± 0.46	7.86 ± 0.72	***p* = 0.001**; ES = −1.00
GPS	6.74 ± 0.41	6.70 ± 0.36	6.81 ± 0.50	*p* = 0.397; ES = −0.26
Timing gates	8.51 ± 1.51	8.15 ± 1.14	9.16 ± 1.88	*p* = 0.20; ES = −0.70
Video analysis	8.46 ± 0.84	8.06 ± 0.60	9.20 ± 0.71	***p* < 0.001**; ES = −1.78
**V0 (m·s^−1^)**				
Linear encoder	8.86 ± 0.61	8.67 ± 0.45	9.19 ± 0.73	***p* = 0.003**; ES = −0.93
GPS	8.63 ± 0.70	8.39 ± 0.46	9.03 ± 0.86	***p* = 0.001**; ES = −1.02
Timing gates	8.41 ± 0.56	8.28 ± 0.49	8.65 ± 0.61	***p* = 0.022**; ES = −0.70
Video analysis	8.15 ± 0.50	8.06 ± 0.43	8.33 ± 0.59	*p* = 0.069; ES = −0.55

V_MAX_ (m·s^−1^) = maximal velocity; P_MAX_ (W·kg^−1^) = maximal power output; F_0_ (N·kg^−1^) = theoretical maximal force; V_0_ (m·s^−1^) = theoretical maximal velocity; bold = significant difference (*p* < 0.05).

## Data Availability

The data presented in the study are not publicly available due to privacy and ethical restrictions. But data are available on request from the corresponding author.
